# Pneumatic Compression Combined with Standard Treatment after Total Hip Arthroplasty and Its Effects on Edema of the Operated Limb and on Physical Outcomes: A Pilot Clinical Randomized Controlled Study

**DOI:** 10.3390/jcm12124164

**Published:** 2023-06-20

**Authors:** Vittoria Carnevale Pellino, Alessandro Gatti, Matteo Vandoni, Pamela Patanè, Massimiliano Febbi, Stefania Ballarin, Caterina Cavallo, Luca Marin

**Affiliations:** 1 Laboratory of Adapted Motor Activity (LAMA), Department of Public Health, Experimental Medicine and Forensic Science, University of Pavia, 27100 Pavia, Italy; vittoria.carnevalepellino@unipv.it (V.C.P.); alessandro.gatti08@universitadipavia.it (A.G.); luca.marin@unipv.it (L.M.); 2 Industrial Engineering Department, University of Tor Vergata, 00133 Rome, Italy; pamela.patane01@universitadipavia.it; 3 Laboratory for Rehabilitation Medicine and Sport (LARMS), 00133 Rome, Italy; massimilianofebbi@gmail.com (M.F.); stefania.ballarin@iacer.it (S.B.); 4 Department of Research, ASOMI College of Sciences, 2080 Marsa, Malta; 5 Luxembourg Health & Sport Sciences Research Institute A.s.b.l., 4530 Luxembourg, Luxembourg; cavallo.caterina@stud.lunex-university.net; 6 Department of Rehabilitation, Istituto di Cura “Città di Pavia”, 27100 Pavia, Italy

**Keywords:** leg pneumatic compression, total hip arthroplasty, lower limb edema

## Abstract

Total hip arthroplasty (THA) is one of the most successful orthopedic procedures and is highly effective at improving function and quality of life. However, patients commonly experience edema immediately after hospitalization and also after discharge, which can lead to health consequences and a lower quality of life. For these reasons, the aim of this study (NCT05312060) was to evaluate the effectiveness of a specific intermittent leg pneumatic compression on lower limb edema and physical outcomes in patients after total hip arthroplasty, compared to standard conservative treatment. A total of 47 patients were enrolled and randomly allocated into two groups: the pneumatic compression group (PG = 24) and the control group (CG = 23). The CG performed the standard venous thromboembolism therapy, which included pharmacological prophylaxis, compressive stockings, and electrostimulation, while the PG combined pneumatic compression with standard VTE therapy. We evaluated thigh and calf circumferences, knee and ankle ranges of motion, pain, and walking autonomy. Our results showed a greater reduction in thigh and calf circumferences for PG (*p* < 0.001), while other outcomes were similar for the two groups (*p* > 0.05). The combination of standard therapy with pneumatic leg compression was more effective at reducing lower limb edema and thigh and calf circumferences than standard treatment. Our results suggest that pressotherapy treatment is a valuable and efficient option for managing lower limb edema after THA.

## 1. Introduction

Total hip arthroplasty (THA) is one of the most successful orthopedic procedures and is highly effective at relieving pain and improving joint function and patients’ quality of life [1,2]. In Italy, THA use has increased over the last 20 years, and this is mainly related to the increase in patients’ longevity (higher number of candidates for the surgery), the higher expectations of patients in terms of quality of life, and the possibility of performing recreational activities again [3]. The complication rate for the THA is quite low, but major complications such as venous thromboembolism (VTE-incidence of 0.6–1.5%) can be highly debilitating, due to delayed hospital discharge and potentially worsened outcomes after the surgery [4]. In fact, a study by O’Reilly et al. reported that after discharge from the hospital, patients had a higher risk of developing VTE, with a prevalence of 8.9% [5]. The risk factors for VTE are described by Virchow’s triad: hypercoagulable state, endothelial injury, and venous stasis, of which at least two factors are generally necessary for VTE development [6]. The endothelial injury, leading to edema development, is inevitable during the surgery, but it can be minimized using proper surgical techniques. The hypercoagulable state is a local and systemic inflammatory response induced by tissue injury during surgery [7,8,9]. Venous stasis, which can occur both during and after surgery, due to the use of tourniquets, prolonged immobilization during the operation, and reduced mobility in the postoperative period, presents a range of potential risks [10]. Among these risks, the development of edema is a notable complication. Edema refers to the abnormal buildup of fluid in the tissues and is characterized by symptoms such as limited range of motion, discomfort, skin changes, and impaired circulation [11]. To effectively address these risks, implementing early mobilization and incorporating rapid recovery programs have demonstrated success in reducing complications following total hip arthroplasty (THA). It is evident, therefore, that a multimodal treatment approach is of utmost importance. This approach may include measures such as employing graduated compression stockings and implementing pharmacologic prophylaxis to inhibit the coagulation cascade and prevent VTE [12,13,14]. The importance of multimodal treatment, such as graduated compression stockings and pharmacologic prophylaxis against VTE by inhibiting the coagulation cascade, is, therefore, evident [12,13,14]. The introduction of other non-pharmacological interventions, such as pneumatic compression (pressotherapy), could lead to a reduced risk of developing VTE, with a better prognosis related to edema resorption and improved physical outcomes. The Cochrane review by Kakkos et al. [15] showed that a combination of intermittent pneumatic leg compression and pharmacological prophylaxis can reduce the incidence of deep vein thrombosis and pulmonary embolism, compared with compression or pharmacological prophylaxis alone [15], but the effectiveness of combined treatments on reducing VTE development remains unclear. Despite these results, all 15 randomized controlled trials included in this review showed a low reliability, due to a high risk of selection and performance biases, highlighting the necessity of further studies to confirm (or not) the effectiveness of combined treatments. A subsequent Cochrane review provided by Zhao et al. [16] evaluated the efficacy of different intermittent pneumatic leg compressions combined with pharmacological treatment in VTE reduction without reaching significant and reliable results, underlining the lack of randomized controlled trials for this topic. In particular, several studies highlighted the persistence of edema in patients after total hip arthroplasty, with a higher risk of VTE development and a decrement in functional capacity and quality of life [17,18,19]. For these reasons, the aim of this preliminary study was to evaluate the effectiveness of a specific intermittent leg pneumatic compression combined with standard treatment on lower limb edema and physical outcomes in patients after total hip arthroplasty, compared to standard conservative treatment.

## 2. Materials and Methods

### 2.1. Study Design and Participants

A pilot randomized controlled trial (NCT05312060) using randomly sequenced blocks of variable sizes was conducted. For the block design elaboration, a generation of random numbers was carried out by the STATA statistical software (release 17.0, 2021, Stata Corporation, College Station, TX, USA), using the “ralloc” command. The researchers were unaware of the block size to avoid the possibility of sequence predictability.

A total of 47 patients from the Department of Orthopedic Surgery of the University Hospital “Istituto di Cura Città di Pavia” (Pavia, Italy) were enrolled, in accordance with the surgeons and the therapists, and were randomly allocated into two groups: the pneumatic compression group (PG = 24) and the control group (CG = 23). The CG performed the standard VTE therapy, which included pharmacological prophylaxis, compressive stockings, and electrostimulation (T-ONE Rehab, I-TECH Medical Division, Scorzè, Italy). The PG combined pneumatic compression with standard VTE therapy.

The inclusion criteria were: elective total hip arthroplasty via the postero-lateral approach, age between 50 and 80 years old, both genders, and the ability to understand the study protocol and procedure. The exclusion criteria were: co-morbidities, such as obesity and diabetes, orthopedic or neurological pathologies that modify the ability to walk, pathologies that modify balance (neurological and/or vestibular), contraindications to the use of the medical equipment used in the study, and inability to understand and sign the informed consent.

The study protocol and procedure were explained to the patients before their engagement in the study, and written informed consent was obtained. Patients could withdraw from the study at any moment without repercussions. The study protocol was approved by the Ethical Committee of Fondazione IRCSS Policlinico San Matteo of Pavia (protocol number 0014626/22) and was performed according to the Declaration of Helsinki as revised in 2018 [20].

### 2.2. Anthropometric Characteristics

Weight was measured by placing the individual in an upright position, wearing light clothing, at the center of a scale platform (Seca, Hamburg, Germany). The individual stood with hands at the sides, looking straight ahead, and facing the person recording the measurements. To determine standing height, a Harpenden stadiometer (Holtain Ltd., Cross-well, Crymych, UK) was utilized. The stadiometer featured a fixed vertical backboard and an adjustable headpiece. Then, body mass index (BMI) was calculated as body weight (kilograms) divided by the height squared (meters squared).

### 2.3. Study Protocol and Evaluation

All patients were enrolled by the surgeon before the THA intervention after the explanation of the study protocol procedure.

All assessments were performed by the same trained specialist 1 day after the THA surgery (T0) and at the end of the treatment (T1). The post-treatment evaluation (T1) was performed after the 10 days of treatment and before the hospital discharge. All the evaluations were performed in the afternoon (4–5:00 or 5–6:00 p.m.) in the same clinical setting.

To evaluate postoperative edema, the following measurements were performed on the operated limb:

- The circumference of the distal third of the thigh (15 cm above the upper border of the patella);
- The circumference of the proximal third of the leg (20 cm above the lateral malleolus);
- The knee flexion range of motion (ROM);
- The ankle dorsiflexion ROM.

The circumferences were taken with a tape measure and the ROM with a manual arthrogoniometer. To ensure the repeatability of the measurements, a permanent surgical marker was used to mark the points at which the circumferences were measured, according to the protocol described above.

The Numeric Rating Scale (NRS), a reliable and validated scale [21,22,23], was used to assess subjective pain. Patients would circle a number between 0 and 10 in accordance with the perceived pain intensity (where zero corresponded to ‘no pain at all’, and the upper limit represented ‘the worst pain ever possible’).

The 20 m walking test (20 mwt) was used to evaluate functional capacity. Patients had to walk along a 20 m straight trail as fast as possible [24] without interruption. Prior to the execution of the test, the specialist explained to the patients the procedure and then showed them how to perform it. The time was taken using a chronograph to record the time (stopwatch W073, SEIKO, Tokyo, Japan), where a lower time showed a better performance. The observers performing the evaluations, including the measurements of thigh and calf circumferences, were blinded, and therefore, they were not aware of the patient’s treatment method (PG vs. CG).

### 2.4. The Pneumatic Compression Therapy

The I-Press^®^ instrument (I-Tech Medical Division, Scorzè, Italy) was used to perform the therapy. The instrument consisted of a pumping system connected to two four-chamber pressure leggings, which were responsible for pumping air through the tubes connected to the different chambers of the leg cuffs. The device reproduced the mechanism of controlled compression on the limbs with a distal–proximal movement (from the periphery towards the center). The instrument features were power supply 230 V; 50 Hertz; current 0,1 A; therapy time 0–30 min; pressure 200 ± 20% mmHg; and weight 2 kg.

The PG performed the pneumatic compression therapy two times per day for a total of 10 days; each session lasted 30 min. To ensure the correct use of the device, the inflatable legging was placed on the operated limb by the physiotherapist, and the pressure was set at 100 mmHg. Prior to the start of the study protocol, the physiotherapist performed two weeks of instrument familiarization to avoid any possible procedure bias.

### 2.5. Statistical Analysis

To ensure the correctness of sample numerosity, a sample size calculation was performed. Pearson’s chi-square test for two independent proportions, considering an alpha error equal to 0.05, a power of 80%, and an effect size equal to 1.76 (according to Cohen’s classification), was performed. All quantitative data were shown as the mean ± standard deviation (SD) if normally distributed or as the median (interquartile range) if not normally distributed. We tested for normality using Shapiro–Wilk tests and graphically checked for linearity. We used an independent samples *t*-test, a parametric Student, or a non-parametric Mann–Whitney as appropriate to evaluate the differences between the pressotherapy and the control group changes. To estimate the size of the effect, we used the Cohen’s d if the data were normally distributed or the rank-biserial correlation if not normally distributed. All the significances were set at a *p*-value less than 0.05. Statistical analyses were performed using the Jamovi Project (2021) (Jamovi Version 1.6 for Mac [Computer Software], Sydney, Australia; retrieved from https://www.jamovi.org (accessed on 5 March 2023)).

## 3. Results

The baseline characteristics of the 47 patients (30 females, 17 males, aged 68.20 ± 9.13 years, range 50–80) enrolled in the study are reported in Table 1.

A comparison between the PG and the CG for all outcomes is shown in Table 2. In particular, PG had a greater reduction in calf circumference (*p* < 0.001) and in thigh circumference (*p* < 0.001) than CG after the administration of the combined protocol.

**Figure 1:**
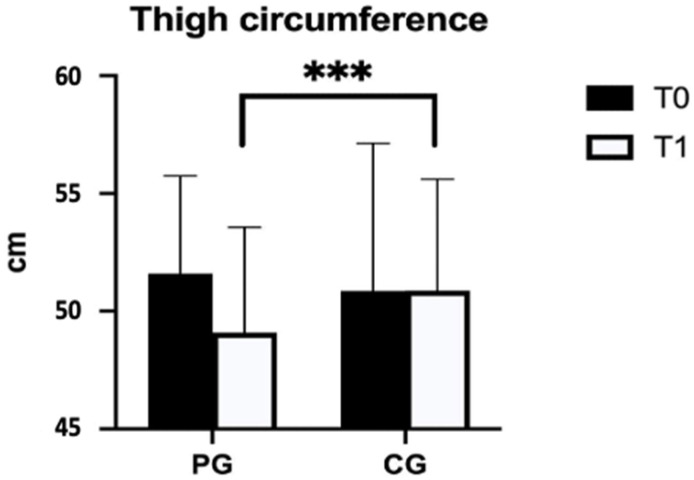
Difference between the PG and the CG in thigh circumference. Legend: PG = pneumatic compression group; CG = control group; cm = centimeters; *** *p* < 0.001.

**Figure 2:**
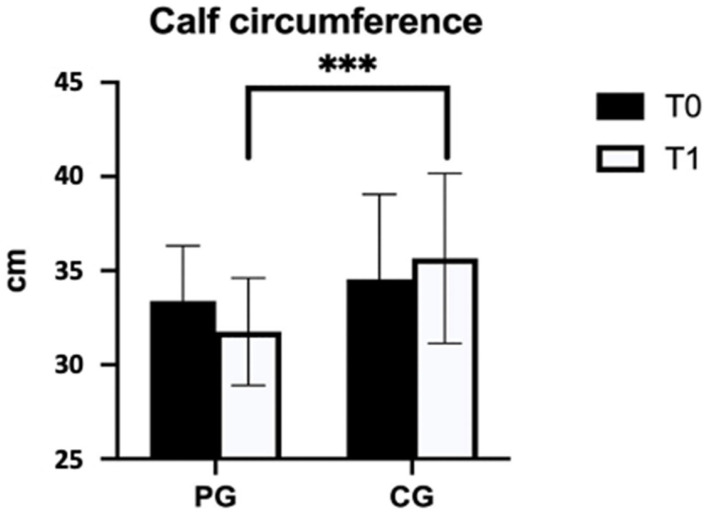
Difference between the PG and the CG in calf circumference. Legend: PG = pneumatic compression group; CG = control group; cm = centimeters; *** *p* < 0.001.

Figure 1 and Figure 2 show the results of PG and CG pre- and post-protocol, underlining the significant differences in thigh and calf circumferences.

## 4. Discussion

The aim of our study was to evaluate the effectiveness of intermittent pneumatic compression combined with standard VTE treatment in reducing edema and improving the physical outcomes of the operated lower limb in patients after THA and to compare it to standard VTE treatment alone. The results of our study showed that the combination of standard therapy with pneumatic leg compression was more effective at reducing thigh and calf circumferences (*p* < 0.001). The two types of treatments (standard and combined treatment) did not differ in improving the range of motions (ROM) of the knee and ankle or in reducing the perceived pain (*p* > 0.05). These results are in accordance with the findings of Fujisawa et al. [25], who demonstrated that leg pneumatic compression could decrease the swelling in the lower limbs seven days after the THA operation. Furthermore, Westrich et al. [26] showed a greater effect of leg pneumatic compression combined with the use of aspirin in reducing lower limb edema after total knee arthroplasty, compared to only using pharmacological treatment. Kwak et al. [27] confirmed that a leg pneumatic compression treatment was more effective at reducing lower limb edema and VTE incidence after THA. The reduction in lower limb edema is an essential part of therapy after THA. Edema can cause various problems, including skin damage, increased risk of infection, and impaired circulation [11], which can lead to the development of one of the three factors of Virchow’s triad (venous stasis). After the surgery, venous stasis increases the risk of developing VTE in THA patients, highlighting the relevance of lower limb edema reduction. This type of edema is defined as vasogenic edema, which is caused by surgical trauma and inflammation that can lead to increased vascular permeability in the surrounding tissues, causing fluid extravasation into the interstitial spaces [28]. Additionally, if there is impaired venous drainage or compromised circulation in the lower limbs after surgery, it can result in venous insufficiency and subsequent edema. In fact, by exerting external pressure on the lower limbs, pneumatic compression devices can aid in improving venous and lymphatic flows [29]. In addition, the use of leg pneumatic compression also has other benefits, such as reducing muscle soreness, aiding in the recovery of injured joints, and improving blood flow. Different studies have demonstrated the positive impact of leg pneumatic compression on blood flow across various populations. For instance, Nose et al. [30] observed enhanced venous blood flow in the soleal and popliteal veins among patients with congestive heart failure. Additionally, Zuj et al. [31] conducted research on healthy adults and corroborated the effectiveness of intermittent pneumatic compression in improving blood flow in the lower limbs. Moreover, our patients at the end of the leg pneumatic compression treatment expressed satisfaction with this treatment (data not reported). As shown by Westrich et al. [26], the efficacy of the leg pneumatic compression treatment is related directly to the compliance of the patients during the treatment, suggesting that another explanation of our results could be related to the high satisfaction after the treatment. Indeed, several studies have consistently shown that using leg pneumatic compression under the supervision of a healthcare professional is more enjoyable than standard therapy and attempting the same treatment without expert guidance in a home setting [32,33]. This enhances the therapy’s effectiveness and helps improve THA’s physical outcomes. Furthermore, we did not find any adverse events in both groups, suggesting that leg pneumatic compression is a safe and effective technique. However, it is fundamental that this device is used with the supervision of healthcare professionals who can determine the appropriate pressure level and treatment duration. To the best of our knowledge, no other studies have investigated the effects of leg pneumatic compression on ROM, NRS, and 20 mwt in patients after THA. Our results did not reveal any differences in ROM, which may be due to the area where the edema was located, being distant from the knee and ankle joints. Moreover, we did not observe any differences in 20 mwt or NRS, which could suggest that the edema experienced post-THA had no influence on walking performance and was not associated with perceived pain.

We recognize that our study had some limitations. Firstly, we did not evaluate the effect of pressotherapy on VTE with the D-dimer blood concentration, which could provide more accurate results about VTE risk. Additionally, the duration of our study protocol was limited; we only analyzed the effect of pressotherapy after 10 days of treatment. Since this was a pilot study, future studies should extend the number of participants and the duration of the protocol and investigate the effect of pressotherapy after months of THA to gain a more comprehensive understanding of the effects.

## 5. Conclusions

The combination of standard therapy with pneumatic leg compression is more effective at reducing thigh and calf circumferences than the standard treatment. Our results suggest that pressotherapy treatment is a valuable and efficient option for managing lower limb edema, but its efficacy on physical outcomes after THA remains unclear. For this reason, future studies should investigate this effect in order to enhance the effectiveness of this treatment after THA. Since we did not find any adverse event after the leg pneumatic compression therapy and for its efficacy on lower limb edema, it is recommended that pneumatic compression of the operated limb should be included in THA’s standard post-operative therapy.

## Figures and Tables

**Table 1:** Descriptive characteristics of the sample.

	PG (*n* = 24)	CG (*n* = 23)	*p*-Value
Male:Female	9:15	8:15	//
Age (years)	68.38 ± 9.35	68.09 ± 9.11	>0.05
Weight (kg)	71.71 ± 12.77	73.57 ± 12.28	>0.05
Height (m)	1.65 ± 0.09	1.68 ± 0.10	>0.05
BMI (kg/m^2^)	25.96 ± 3.14	25.72 ± 2.59	>0.05

All data are shown as mean ± SD. Legend: kg = kilograms; m = meters; BMI = body mass index.

**Table 2:** Comparisons of measurement changes at baseline (T0) and after the intervention (T1) in the PG and CG.

		T0	T1	Delta (Post-Pre)	*p*-Value	Effect Size
Thigh circumference (cm)	PG (*n* = 24)	51.6 ± 4.1	49.1 ± 4.5	−2.0 (2.0)	<0.001 ***	0.850
	CG (*n* = 23)	50.9 ± 6.3	50.9 ± 4.7	0.2 (1.4)		
Calf circumference (cm)	PG (*n* = 24)	33.4 ± 2.9	31.8 ± 2.9	−1.9 (1.6)	<0.001 ***	0.690
	CG (*n* = 23)	34.5 ± 4.5	35.7 ± 4.5	0.0 (0.7)		
Knee ROM (°)	PG (*n* = 24)	99.38 ± 23.95	117.20 ± 9.97	12.5 (14.0)	0.528	//
	CG (*n* = 23)	93.91 ± 23.95	110.57 ± 12.09	15 (11.00)		
Ankle ROM (°)	PG (*n* = 24)	91.79 ± 4.37	95.58 ± 6.07	4.00 (6.00)	0.966	//
	CG (*n* = 23)	87.96 ± 6.26	92.09 ± 3.90	3.00 (7.00)		
NRS	PG (*n* = 24)	4.92 ± 2.00	2.46 ± 1.61	−2.50 (1.00)	0.697	//
	CG (*n* = 23)	6.13 ± 2.24	4.00 ± 1.73	−2.00 (1.00)		
20 mwt (s)	PG (*n* = 24)	52.44 ± 22.79	25.27 ± 4.83	−15.50 (23.75)	0.983	//
	CG (*n* = 23)	58.39 ± 20.62	34.52 ± 10.24	−23.00 (21.00)		

All data are shown as the mean ± SD or as the median (interquartile range); *** *p* < 0.01 as significant values. Legend: PG = pneumatic compression group; CG = control group; cm = centimeters; ° = grade; ROM = range of motion; NRS = Numeric Rating Scale; mwt = meter walking test.

## Data Availability

The data presented in this study are available on request from the corresponding author. The data are not publicly available due to privacy reasons.
